# Comprehensive Analysis of N6-Methyladenosine RNA Methylation Regulators Expression Identify Distinct Molecular Subtypes of Myocardial Infarction

**DOI:** 10.3389/fcell.2021.756483

**Published:** 2021-10-27

**Authors:** Xin Shi, Yaochen Cao, Xiaobin Zhang, Chang Gu, Feng Liang, Jieyuan Xue, Han-Wen Ni, Zi Wang, Yi Li, Xia Wang, Zhaohua Cai, Berthold Hocher, Ling-Hong Shen, Ben He

**Affiliations:** ^1^Department of Cardiology, Shanghai Chest Hospital, Shanghai Jiao Tong University, Shanghai, China; ^2^Department of Nephrology, Charité-Universitätsmedizin Berlin, Berlin, Germany; ^3^Department of Cardiovascular Surgery, Shanghai Chest Hospital, Shanghai Jiao Tong University, Shanghai, China; ^4^Department of Thoracic Surgery, Shanghai Pulmonary Hospital, Tongji University School of Medicine, Shanghai, China; ^5^5th Department of Medicine (Nephrology, Hypertensiology, Endocrinology, Rheumatology), University Hospital Mannheim, University of Heidelberg, Mannheim, Germany

**Keywords:** myocardial infarction, RNA methylation, m6A RNA modification, PPI network, DEG analysis

## Abstract

**Background:** Myocardial infarction (MI) is one of the leading threats to human health. N6-methyladenosine (m6A) modification, as a pivotal regulator of messenger RNA stability, protein expression, and cellular processes, exhibits important roles in the development of cardiac remodeling and cardiomyocyte contractile function.

**Methods:** The expression levels of m6A regulators were analyzed using the GSE5406 database. We analyzed genome-wide association study data and single-cell sequencing data to confirm the functional importance of m6A regulators in MI. Three molecular subtypes with different clinical characteristics were established to tailor treatment strategies for patients with MI. We applied pathway analysis and differentially expressed gene (DEG) analysis to study the changes in gene expression and identified four common DEGs. Furthermore, we constructed the protein–protein interaction network and confirmed several hub genes in three clusters of MI. To lucubrate the potential functions, we performed a ClueGO analysis of these hub networks.

**Results:** In this study, we identified that the levels of FTO, YTHDF3, ZC3H13, and WTAP were dramatically differently expressed in MI tissues compared with controls. Bioinformatics analysis showed that DEGs in MI were significantly related to modulating calcium signaling and chemokine signaling, and m6A regulators were related to regulating glucose measurement and elevated blood glucose levels. Furthermore, genome-wide association study data analysis showed that WTAP single-nucleotide polymorphism was significantly related to the progression of MI. In addition, single-cell sequencing found that WTAP is widely expressed in the heart tissues. Moreover, we conducted consensus clustering for MI in view of the dysregulated m6A regulators’ expression in MI. According to the expression levels, we found MI patients could be clustered into three subtypes. Pathway analysis showed the DEGs among different clusters in MI were assigned to HIF-1, IL-17, MAPK, PI3K-Akt signaling pathways, etc. The module analysis detected several genes, including BAG2, BAG3, MMP2, etc. We also found that MI-related network was significantly related to positive and negative regulation of angiogenesis and response to heat. The hub networks in MI clusters were significantly related to antigen processing and ubiquitin-mediated proteolysis, RNA splicing, and stability, indicating that these processes may contribute to the development of MI.

**Conclusion:** Collectively, our study could provide more information for understanding the roles of m6A in MI, which may provide a novel insight into identifying biomarkers for MI treatment and diagnosis.

## Introduction

Myocardial infarction (MI) is referred to as a heart attack event term in which myocardial cells die because of imbalances between oxygen supply and demand. The general definition of MI consists of five subtypes, among which type 1 and type 2 MI are the widely occurred sorts and also exhibit a tight relationship to clinicians (Gao et al., 2016). Type 1 MI is induced by acute atherosclerotic thrombosis, whereas type 2 MI results from the imbalanced supply and demand of myocardial oxygen without acute atherosclerotic thrombosis (Lu et al., 2015; Sandoval and Jaffe, 2019). The most common cause of MI is blood flow to a part of the heart is reduced or paused, leading to myocardial necrosis. Also, the result of a blood coagulum in the coronary artery is responsible for supplying the heart muscle area. The risk of MI usually increases with age, particularly at the age of more than 65 years (Lu et al., 2015).

Previous research has shown that epigenetic regulation plays importantly in the regulation of cardiovascular repair functions. Typically, as the ubiquitous and abundant transcription modification of messenger RNA (mRNA) and long non-coding RNA in the genome of eukaryotes, N6-methyladenosine (m6A) primarily occurs in the 3′-untranslated regions and nearby the stop codons of mRNAs (Meyer et al., 2012; Wang et al., 2014; Li et al., 2017; Liu et al., 2017; Zhang et al., 2017). Acting as a reversible modification, m6A is methylated by m6A methyltransferases (writers), demethylated by m6A demethylases (erasers), and recognized by m6A binding proteins (readers), which participates in various biological processes. Dysregulated m6A is gradually thought to be the etiopathogenesis of certain diseases, such as carcinomas and cardiovascular disease (CVD). Growing evidence indicated that the continuous dynamic modulation of m6A exerts an impact on specific genes’ expression and some diseases’ physiological and pathological processes, including MI, ischemic heart failure (HF), myocardial hypertrophy, and cardiomyogenesis (Mathiyalagan et al., 2019). For instance, WTAP promotes MI *via* modulating m6A modification of ATF4 mRNA (Wang et al., 2021). Dorn et al. (2019) showed that N6 adenosine methylation regulated by METTL3 is crucial for hypertrophy’s pathological process *in vivo* and *in vitro*. Loss of METTL3 enhanced heart regeneration and repair after myocardial injury (Gong et al., 2021). Nonetheless, the roles of m6A regulators in MI remained largely unclear.

So far, the function of m6A in physiological and biological processes has been largely studied. Nevertheless, the research toward m6A is limited under pathological conditions in MI. Here, we systematically investigated the role of m6A epigenetic regulation in MI. Furthermore, we applied unsupervised consensus clustering on gene expression in MI based on m6A expression. In addition, we identified the potential roles of differentially expressed genes (DEGs) in different clusters based on m6A expression by performing Kyoto Encyclopedia of Genes and Genomes (KEGG) analysis and protein–protein interaction (PPI) network analysis. As far as we know, our literature, for the first time, comprehensively analyzed m6A’s roles in MI, which may provide novel clues to identify biomarkers for MI treatment and diagnosis.

## Materials and Methods

### Data Collection

GSE5406 (Hannenhalli et al., 2006) included gene expression profile of human left ventricle tissue from 16 non-failing myocardium samples, 108 ischemic myocardium samples, and 86 idiopathic myocardium samples. Genome-wide association study (GWAS) data were downloaded from the GeneAtlas database.^1^ The single-cell data were from The Single Cell Type Atlas.

### Enrichment Analysis

We carried out functional enrichment analysis, including Gene Ontology (GO) and KEGG, using the DAVID system.^2^ *P*-value < 0.05 meant significant difference.

### Construction of Protein–Protein Interaction Network

The STRING database was utilized to construct the PPI network. The PPI network was displayed by Gephi software. We chose the hub genes based on the extent of genes’ connectivity.

### Unsupervised Consensus Clustering Analysis

We carried out unsupervised consensus analysis utilizing the ConsensusClusterPlus (Wilkerson and Hayes, 2010) R package. Taken briefly, the consistent matrix plots were shown in the light of the *k*-value. Also, empirical cumulative distribution function plots exhibited the uniform distributions for each k. What is more, the item tracking plot demonstrated the consistent clustering of items (columns) at each k (rows) to determine the clustering stability. The cluster-consensus plot illustrated the cluster-consensus value at different *k*-values. High cluster-consensus value meant clustering with low stability. Item-consensus plot is the mean consensus value deriving from an item and a consensus cluster’s members. An item indicated several item-consensus values with different ks.

### Differentially Expressed Gene Analysis

The DEGs were determined by the Limma R package between different clusters in AMI, observing the cutoff value of | log2 (fold change) | ≥ 1 and *P*-value < 0.05. Ggplot R package was applied to draw the volcano plot, and pheatmap R package was used to plot the heatmap of DEGs.

### Potential Hub Gene Identification

Each protein node’s degree was evaluated by the CytoHubba plugin in Cytoscape software^3^ (Chin et al., 2014; Ma et al., 2021). Nodes with a higher degree of connectivity are normally more important for keeping the entire network’s stability. The 10 proteins with closet connection nodes here were the potential hub genes.

## Results

### N6-Methyladenosine Regulators Expression Was Dysregulated in Myocardial Infarction

**FIGURE 1:**
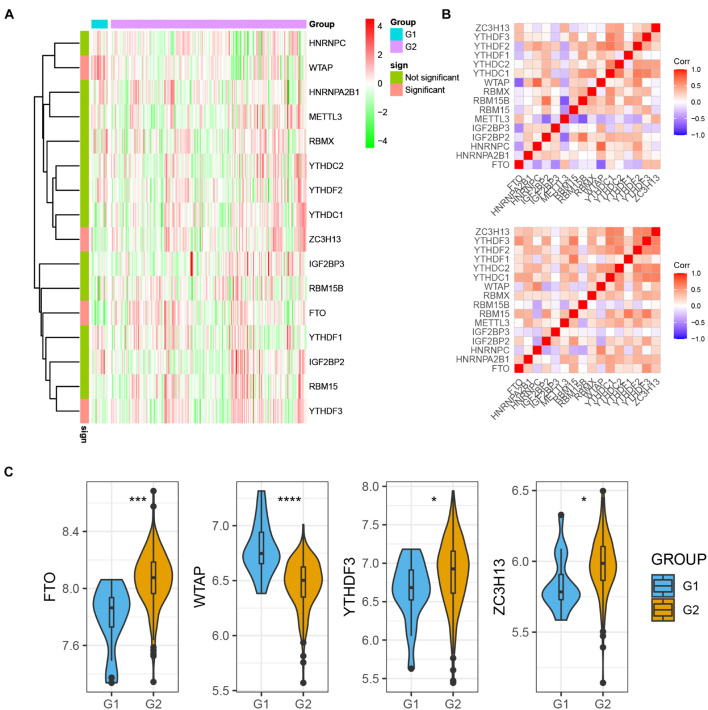
m6A regulators expression was dysregulated in MI. **(A)** m6A modulators were differently expressed in MI. **(B)** Correlation among m6A regulators’ expression in MI samples and non-failing samples. **(C)** Dysregulation of FTO, YTHDF3, ZC3H13, and WTAP in MI was presented. *P < 0.05; ***P < 0.001; ****P < 0.0001.

For assessing m6A regulators’ biological function in MI’s occurrence and development, we explored 15 m6A regulators’ expression profiles systematically between MI and non-failing controls using GSE5406. Figure 1A illustrates m6A modulators’ expression levels in MI and non-failing controls separately. Among them, we observed that FTO, YTHDF3, and ZC3H13 expression levels were largely lower in MI tissues than in non-failing controls (*p* < 0.01); however, WTAP expression levels were markedly higher in MI tissues relative to non-failing controls (*p* < 0.05). In addition, there was no obvious difference between the non-failing controls and MI tissues with regard to RBM15, YTHDC1, HHRNPA2B1, IGF2BP2, METTL3, RBMX, YTHDF1, YTHDF2, HNRNPC, IGF2BP3, RBM15B, and YTHDC2 expression levels. To evaluate whether m6A modulators functioned crucially in the development of MI, we analyzed the correlation among them in non-failing controls and MI samples separately. The results demonstrated that the correlation among m6A regulators has a significant change between control and MI groups, implying that m6A modulators functioned crucially in the development of MI (Figure 1B). The dysregulation of FTO, YTHDF3, ZC3H13, and WTAP in MI is presented in Figure 1C.

### Functional Annotation of N6-Methyladenosine Regulators in Myocardial Infarction

**FIGURE 2:**
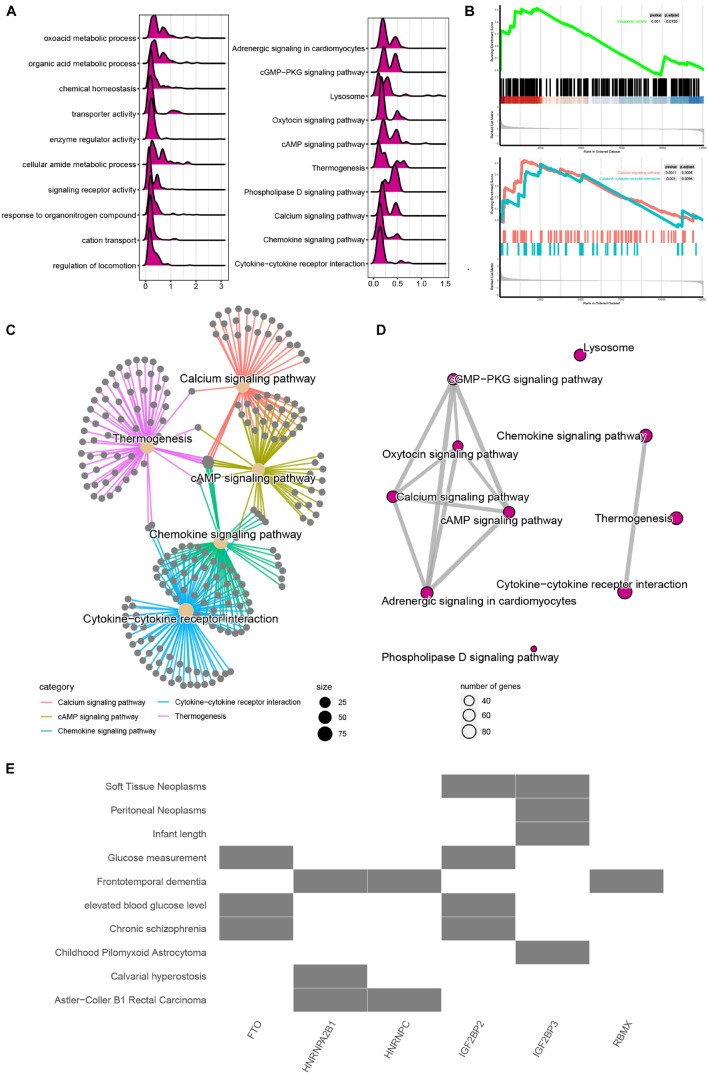
Functional annotation of m6A regulators in MI. **(A)** Mountain map presented functional annotation of DEGs in MI. **(B)** Gene Set Enrichment Analysis demonstrated that DEGs in MI were related to transport activity and calcium signaling. **(C)** Top 5 enriched signaling have strong crosstalk. **(D)** Calcium signaling and chemokine signaling play a key role in MI. **(E)** Functional annotation of m6A regulators in MI.

To evaluate the potential functions of m6A regulators in MI, we performed bioinformatics analysis. As present in Figure 2A, the mountain map shows that the DEGs in MI were significantly enriched in regulation of locomotion, cation transport, response to organonitrogen compound, signaling receptor activity, cellular amide metabolic process, enzyme regulator activity, transporter activity, chemical homeostasis, oxoacid metabolic process, cytokine–cytokine receptor interaction, chemokine signaling, calcium signaling, phospholipase D signaling, thermogenesis, cAMP signaling, oxytocin signaling, lysosome, cGMP-PKG signaling, and adrenergic signaling in cardiomyocytes. Of note, Gene Set Enrichment Analysis further demonstrated that DEGs in MI were related to transport activity and calcium signaling (Figure 2B). Moreover, by analyzing the relationship among the top 5 enriched signalings, we observed these pathways have strong crosstalk (Figure 2C). Calcium signaling and chemokine signaling play a key role in MI (Figure 2D).

We also predicted that the potential signaling related to m6A regulators and observed FTO and IGFBP2 are mainly related to glucose measurement, elevated blood glucose level, and chronic schizophrenia. HNRNPA2B1 and HNRNPC are mainly related to Astler–Coller B1 rectal carcinoma. IGF2BP3 was related to soft tissue neoplasms and peritoneal neoplasms (Figure 2E).

### Confirmation of the Functional Importance of N6-Methyladenosine Regulators in Myocardial Infarction

**FIGURE 3:**
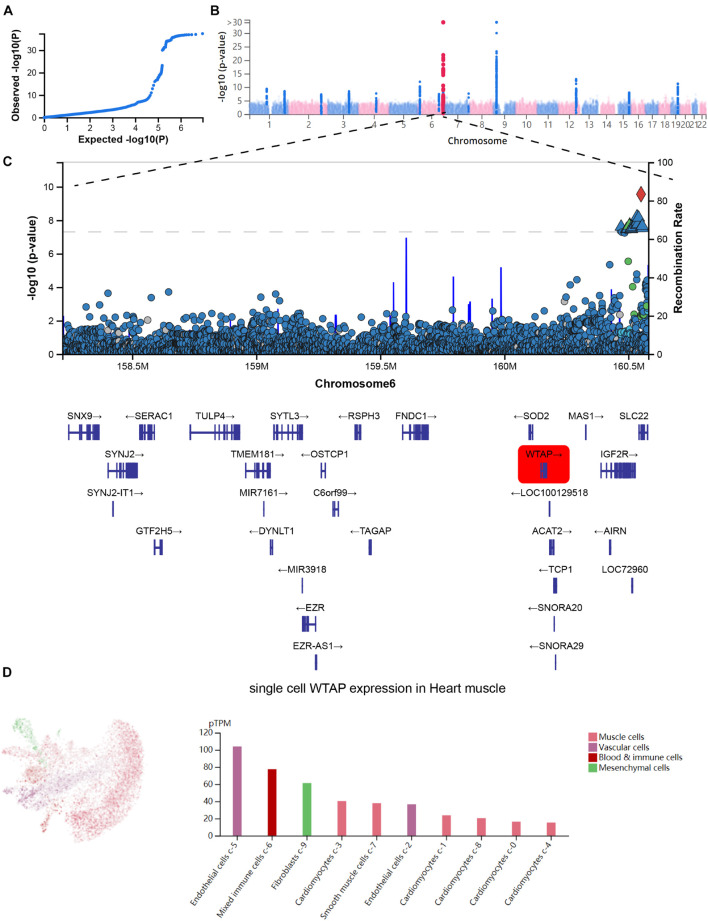
Confirmation of functional importance of m6A regulators in MI. **(A)** Q-Q plot shows that significantly related SNP sites could be identified in GWAS data. **(B)** Manhattan plot representing meta-GWAS results. **(C)** SNP annotation of locus found that m6A regulatory gene WTAP was located in causal region on chromosome 6. **(D)** Single-cell sequencing of heart tissue showed that WTAP is widely expressed in heart tissues.

Next, we analyzed the GWAS data and single-cell sequencing data to confirm the functional importance of m6A regulators in MI. As present in Figure 3A, the Q-Q plot shows that the GWAS data can identify significantly related single-nucleotide polymorphism (SNP) sites. Through precise positioning of the GWAS data, causal SNPs are mainly distributed in the enriched area (Figure 3B). The SNP annotation of the locus found that the m6A regulatory gene WTAP was located in the causal region on chromosome 6, suggesting it may be an important pathogenic gene of MI (Figure 3C). Furthermore, we analyzed the single-cell sequencing of the heart tissues and found that WTAP is widely expressed in the heart tissues, whose expression in endothelial cells is the highest (Figure 3D). By combining the analysis mentioned earlier that WTAP was significantly lower in the MI group, this suggests that maintaining the normal expression of WTAP may be a prevention or improvement means for the treatment of MI.

### Consensus Clustering Analysis for Myocardial Infarction Based on the Expression of N6-Methyladenosine Regulators

m6A RNA methylation exerted an important effect on modulating the mRNA stability (Wang et al., 2014), alternative splicing, and RNA structure (Liu et al., 2017), thus affecting the RNA expression of the human genome. Thus, we performed consensus clustering for MI in view of the dysregulated m6A modulators in MI.

**FIGURE 4:**
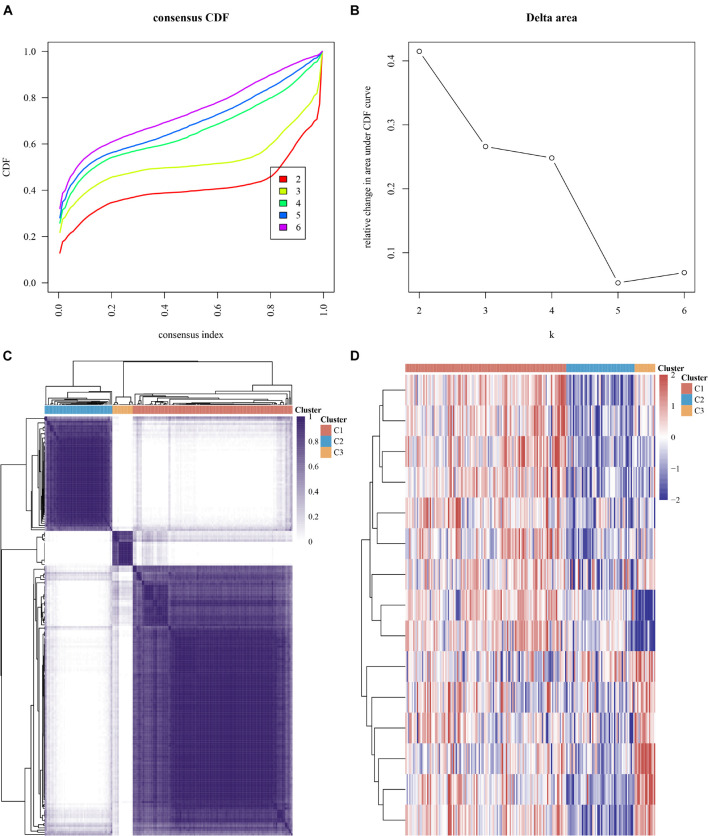
Consensus clustering analysis of MI samples based on mRNA levels of m6A regulators. **(A)** Cumulative distribution function of clustering (k, 2–6). **(B)** Delta area score displayed relative growth in cluster stability. **(C)** Consistency matrix of sample with *k* = 3. **(D)** Heatmap of expression of m6A regulators in three types of samples.

We conducted the consensus clustering utilizing the Consensus Cluster Plus R package. The cumulative distribution function presented the lowest range ability at consensus index 0.2–0.6 with *k* = 3 (Figure 4A). The delta area scores of 2.5 were the highest at *k* = 3 (Figure 4B). Totally, 499 MI patients were clustered into three subtypes, including cluster1 (*n* = 125), cluster2 (*n* = 53), and cluster 3 (*n* = 16) on the basis of m6A regulators expression levels (Figure 4C).

Our data showed that the clustering subtypes defined by m6A regulator expression exhibited a close relation to MI patients’ heterogeneity. To uncover the interplay between m6A regulators, we explored the correlation among 15 m6A RNA methylation regulators. FTO, YTHDF1, IGF2BP3, RBM15B, YTHDF3, IGF2BP2, and RBM15 were mostly upregulated in cluster 3 (Figure 4D). WTAP, HNRNPC, YTHDF2, YTHDC2, HNRNPA2B1, METTL3, ZC3H13, and YTHDC1 were most greatly upregulated in cluster 1 (Figure 4D). Of note, we observed that WTAP and HNRNPC were greatly downregulated in cluster 3 (Figure 4D).

### Identifying the Differentially Expressed Genes Between Different Clusters in Myocardial Infarction

**FIGURE 5:**
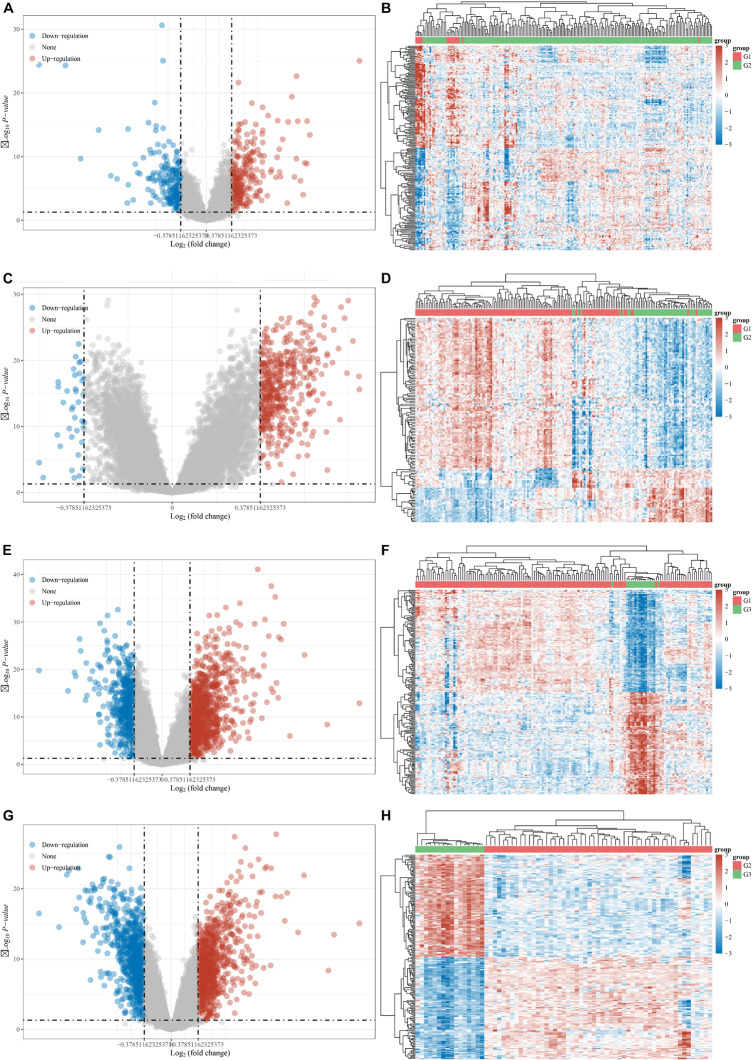
Identification of DEGs between different clusters in MI. **(A,B)** Volcano plot and heatmap showed DEGs in MI compared with non-failing controls. **(C,D)** Volcano plot and heatmap showed DEGs in cluster 1 MI compared with cluster 2 MI samples. **(E,F)** Volcano plot and heatmap showed DEGs in cluster 1 MI compared with cluster 3 MI samples. **(G,H)** Volcano plot and heatmap showed DEGs in cluster 2 MI compared with cluster 3 MI samples.

The DEGs among cluster 1, cluster 2, and cluster 3 MI samples were identified based on m6A regulators expression in depth. Four hundred thirty-seven DEGs were identified in total, including 237 DEGs with upregulation and 200 DEGs with downregulation between MI and non-failing controls (Figures 5A,B). The top 10 overexpressed and suppressed genes between MI and non-failing controls are listed in Table 1. Totally, 456 DEGs, comprising 420 DEGs with upregulation and 36 DEGs with downregulation, were screened after comparing cluster 1 with cluster 2 samples (Figures 5C,D). The top 10 overexpressed and suppressed between cluster 1 and cluster 2 are listed in Table 2. One thousand three hundred ninety-five DEGs in total, consisting of 884 DEGs with upregulation and 511 DEGs with downregulation, were obtained after comparing cluster 1 with cluster 3 samples (Figures 5E,F). The top 10 overexpressed and suppressed genes between cluster 1 and cluster 3 are listed in Table 3. One thousand six hundred seventy-eight DEGs were acquired, including 886 DEGs with upregulation and 792 DEGs with downregulation after comparing cluster 2 with cluster 3 samples (Figures 5G,H). The top 10 overexpressed and suppressed genes between cluster 2 and cluster 3 are listed in Table 4.

**TABLE 1:** Top 10 upregulated and downregulated genes between MI and non-failing controls are listed.

Gene	logFC	AveExpr	*P.*value	adj.P.Val
ASPN	−2.48256	8.496551	3.87E-25	1.2E-21
LUM	−2.08986	8.320778	4.89E-25	1.21E-21
NPPA	−1.86529	10.8008	2.02E-10	2.66E-08
MXRA5	−1.59757	7.934672	6.53E-15	3E-12
HBB	−1.41654	10.49883	9.98E-08	5.51E-06
HBA1	−1.32304	10.09423	3.08E-07	1.33E-05
RPS4Y1	−1.17099	9.009555	0.001842	0.016078
MATN2	−1.16118	6.314503	4.6E-15	2.37E-12
COL1A2	−1.15778	8.924507	2.6E-06	8.18E-05
EIF1AY	−1.08342	6.726727	0.000613	0.006978
CYP4B1	1.338434	5.688301	2.05E-10	2.67E-08
SERPINA3	1.340841	7.140782	2.29E-23	4.73E-20
CNN1	1.360707	8.182115	2.68E-16	2.22E-13
PTX3	1.399252	3.907297	5.87E-07	2.26E-05
NRAP	1.438914	8.433394	9.41E-05	0.00159
ANKRD2	1.464661	8.685692	9.65E-10	1.02E-07
SERPINE1	1.49643	6.6758	1.25E-09	1.27E-07
HOPX	1.498253	6.581294	2.53E-16	2.22E-13
FKBP5	1.539755	5.867897	3.85E-14	1.4E-11
MYOT	2.275057	6.115552	9.34E-26	3.86E-22

**TABLE 2:** Top 10 upregulated and downregulated genes between cluster 1 and cluster 2 are listed.

Gene	logFC	AveExpr	*P*.value	adj.P.Val
C1QA	−0.571568507	8.216145512	3.04084E-05	7.72562E-05
HSPB6	−0.553479972	10.21379282	0.005533761	0.009419184
PLD3	−0.493032521	8.276264223	1.8848E-09	9.96561E-09
GNAI2	−0.48900018	8.44807505	1.02338E-16	2.39208E-15
BBC3	−0.488913721	7.597093426	1.87007E-17	5.20383E-16
CD74	−0.480404826	10.03045673	1.02991E-07	4.01048E-07
ELAVL3	−0.467056891	7.908424731	2.39457E-14	3.19526E-13
MLXIPL	−0.450943739	7.670774042	4.3036E-16	8.69356E-15
TCF15	−0.441620165	7.787716012	2.07934E-10	1.30438E-09
TNFRSF12A	−0.430682917	8.141291884	3.9056E-09	1.94776E-08
UBA3	0.693735064	6.633076354	2.41672E-25	1.19231E-22
HAT1	0.714271162	5.164562065	1.61059E-22	2.59012E-20
MYLK3	0.720861637	6.846852685	9.61771E-12	7.66878E-11
HNRNPH2	0.72567453	6.524209837	8.68335E-29	1.53609E-25
PSMC6	0.740436344	6.349662243	4.16543E-27	4.29837E-24
FBXO3	0.750607311	6.327430267	8.63804E-19	3.51858E-17
UBQLN2	0.756248925	6.660653853	8.99102E-30	3.50471E-26
ERBIN	0.776206403	6.53634623	1.12775E-23	2.79299E-21
TOB1	0.802543982	7.143614217	2.57942E-18	8.84792E-17
AGL	0.80408328	7.161402592	2.50455E-16	5.33039E-15

**TABLE 3:** Top 10 upregulated and downregulated genes between cluster 1 and cluster 3 are listed.

Gene	logFC	AveExpr	*P*.value	adj.P.Val
SCD5	−1.669762217	7.227090618	1.61165E-20	1.3956E-18
NOTCH2NLA	−1.275728669	7.336681086	2.98346E-16	9.213E-15
ZNF160	−1.188776239	7.209424932	4.07997E-20	3.19761E-18
FBXW12	−1.170983809	7.006029389	2.74477E-19	1.71659E-17
HAUS2	−1.118509198	7.733329883	3.59245E-27	1.48284E-24
NDUFS6	−1.103093937	9.24772935	1.23989E-24	2.74171E-22
TUBA4A	−1.039597639	8.545693058	2.70101E-14	5.54669E-13
NOP53	−1.028008056	10.46525204	4.46304E-22	5.63937E-20
ZNF721	−1.019622161	6.552467843	3.02137E-15	7.71416E-14
DENR	−0.977105459	6.848251924	1.08654E-18	6.08806E-17
CLDND1	1.536050305	6.508038296	5.28245E-36	2.18042E-32
PERP	1.552850114	6.963745419	6.59367E-18	2.95831E-16
SYNE2	1.575767781	7.537980983	8.84218E-15	2.03141E-13
PJA2	1.583501558	7.07586907	1.60007E-27	7.07633E-25
CAVIN2	1.61399586	6.797908231	4.74363E-27	1.83564E-24
AIDA	1.649735394	5.820592107	2.49104E-30	2.05643E-27
HSPB6	1.73449924	9.852169996	1.00411E-06	4.25234E-06
GDE1	1.934081045	7.791642574	8.64685E-24	1.62233E-21
NRAP	2.240938729	8.143871183	3.91497E-09	2.69178E-08
PDK4	2.677741722	7.665580196	1.25003E-13	2.25973E-12

**TABLE 4:** Top 10 upregulated and downregulated genes between cluster 2 and cluster 3 are listed.

Gene	logFC	AveExpr	*P*.value	adj.P.Val
SCD5	−1.856635647	7.281264833	3.28662E-17	3.04334E-15
NOTCH2NLA	−1.574205738	7.258473695	2.86831E-15	1.59244E-13
ZNF160	−1.465900073	7.137323362	1.03506E-22	7.53951E-20
DENR	−1.436186968	6.611322062	2.58322E-23	2.13254E-20
FBXW12	−1.331571212	7.021334527	7.78763E-18	8.92909E-16
BTBD1	−1.304062092	8.119054045	1.25244E-23	1.13211E-20
MKNK2	−1.292164426	8.509136599	1.71603E-19	3.5416E-17
MYLK3	−1.23701779	6.627474331	4.11533E-13	1.23634E-11
HOMER1	−1.235259587	7.62399883	2.76164E-17	2.63057E-15
ZNF721	−1.220068053	6.519234476	1.48872E-16	1.19707E-14
CAVIN2	1.467009524	6.49389424	1.21281E-19	2.63477E-17
ARF1	1.477205791	8.464844564	1.75823E-28	2.17722E-24
GPX3	1.545046294	10.64617013	1.38516E-19	2.90719E-17
FHL1	1.596455104	10.23344407	3.71549E-16	2.72242E-14
TXNIP	1.629366083	10.34122111	3.8799E-20	9.80507E-18
HLA-DRA	1.783828972	8.963740875	1.65982E-14	7.39335E-13
GDE1	1.866759022	7.510919083	1.2535E-22	8.62335E-20
PDK4	2.208572305	6.988135287	4.32783E-09	4.51868E-08
HSPB6	2.287979212	10.07192667	3.27511E-14	1.34737E-12
NRAP	2.645117254	8.188980106	8.31601E-16	5.53641E-14

### Bioinformatics Analysis of the Differentially Expressed Genes Between Different Clusters in Myocardial Infarction

**FIGURE 6:**
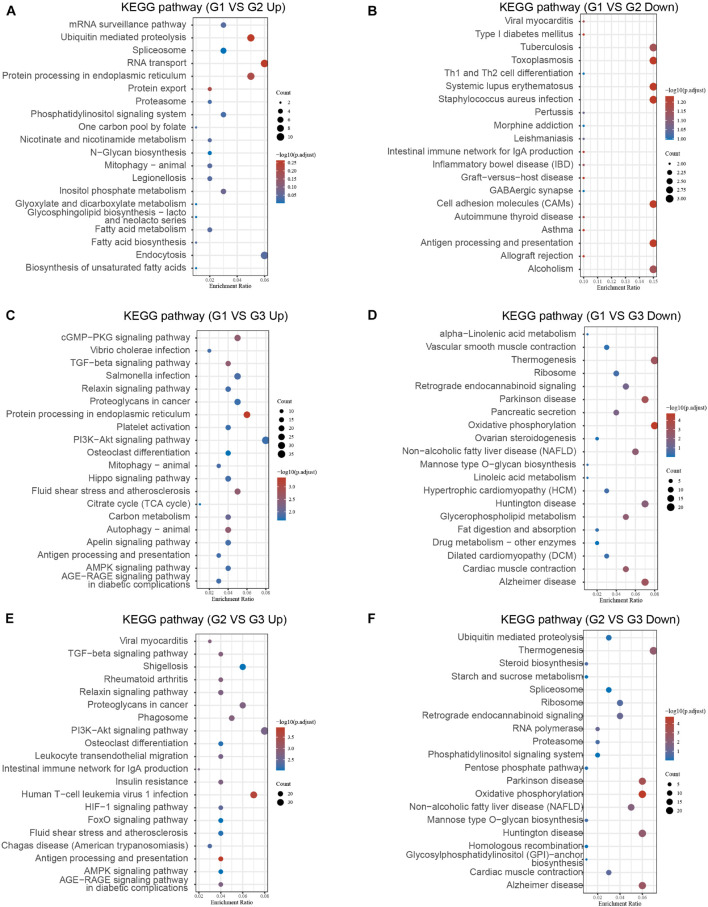
Bioinformatics analysis of DEGs between different clusters in MI. **(A–F)** KEGG analysis revealed potential signaling regulated by upregulated DEGs and downregulated genes in cluster 1 MI compared with cluster 2 MI samples **(A,B)**, in cluster 1 MI compared with cluster 3 MI samples **(C,D)**, and in cluster 2 MI compared with cluster 3 MI samples **(E,F)**.

We further performed KEGG analysis of DEGs between different clusters to evaluate their potential functions in MI. As present in Figure 4, the overexpressed genes in cluster 1 compared with cluster 2 were significantly related to fatty acid biosynthesis, RNA transport, ubiquitin-mediated proteolysis, protein processing (Figure 6A). After comparison with cluster 2, the downregulated genes in cluster 1 were significantly related to toxoplasmosis, cell adhesion molecules, antigen processing and presentation, and alcoholism (Figure 6B). In comparison with cluster 3, the overexpressed genes in cluster 1 were significantly related to antigen processing and presentation, autophagy, and signaling pathways, including AGE-RAGE, AMPK, PI3K-Akt, TGF-beta, and cGMP-PKG signaling (Figure 6C). After comparison with cluster 3, the overexpressed genes in cluster 2 were significantly related to antigen processing and presentation and signaling pathways, including AGE-RAGE, AMPK, FoxO, HIF-1, PI3K-Akt, and TGF-beta signaling (Figure 6E). The downregulated genes in cluster 1 compared with cluster 3 and cluster 2 compared with cluster 3 exhibited a significant relation to oxidative phosphorylation, spliceosome, and multiple neurodegenerative diseases, such as Alzheimer’s disease, Huntington’s disease, and Parkinson’s disease (Figures 6D–F).

### Screening of Key Genes in Different Clusters of Myocardial Infarction

**FIGURE 7:**
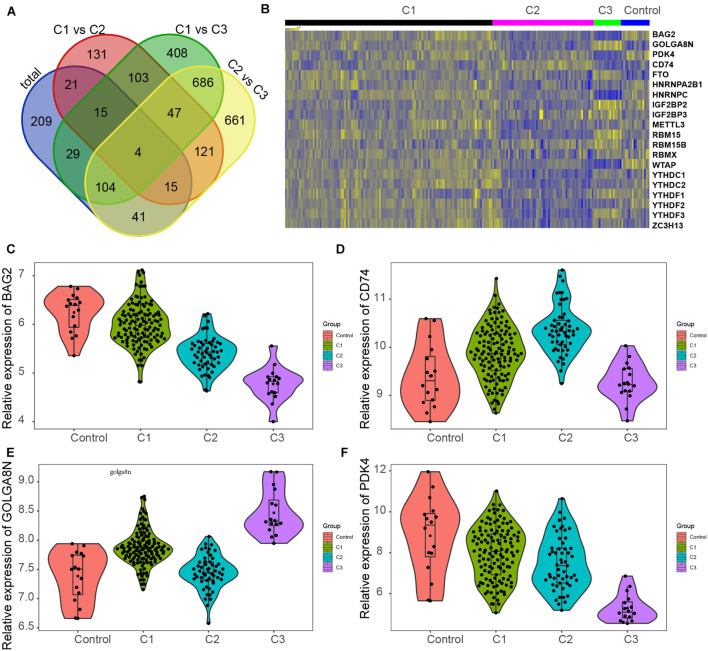
Screening of key genes in different clusters of MI. **(A)** Venn diagram showed four common DEGs among all clusters in MI, including BAG2, CD74, GOLGA8N, and PDK4. **(B)** Heatmap showed BAG2, CD74, GOLGA8N, and PDK4 expression among all clusters in MI. **(C–F)** BAG2 **(C)**, CD74 **(D)**, GOLGA8N **(E)**, and PDK4 **(F)** were differently expressed among all clusters in MI.

We also investigated changes in gene expression among different clusters in MI based on the expression change of the GSE5406 dataset. As present in Figure 6, we observed that approximately 45.7% of DEGs were differently expressed in more than two different clusters (Figure 7A). Venn diagram showed that there were four common DEGs among all clusters in MI, including BAG2, CD74, GOLGA8N, and PDK4 (Figure 7B).

The results showed that BAG2 and PDK4 have a similar expression pattern in MI, which were suppressed in all clusters of MI compared with non-failing controls, suppressed in cluster 2 in comparison with cluster 1 samples, and suppressed in cluster 3 after comparison with cluster 2 and cluster 1 samples (Figures 7C,F). Meanwhile, we found that CD74 was enhanced in clusters 1 and 2 after comparison with non-failing controls, enhanced in cluster 2 in comparison with cluster 1 samples, but suppressed in cluster 3 relative to cluster 1 and 2 samples (Figure 7D). Finally, the results showed that GOLGA8N was enhanced in clusters 1 and 3 in comparison with non-failing controls but suppressed in cluster 2 in comparison with cluster 1 samples and enhanced in cluster 3 in comparison with cluster 1 and 2 samples (Figure 7E).

### Screening of Hub Networks Between Different Clusters of Myocardial Infarction

**FIGURE 8:**
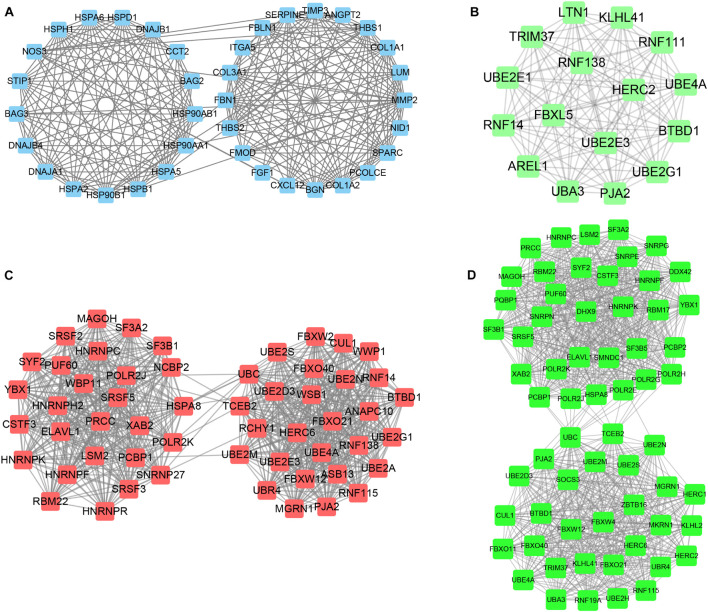
Identification of hub networks in different clusters in MI. **(A–D)** We constructed hub networks based on DEGs in MI compared with non-failing controls **(A),** in cluster 1 MI compared with cluster 2 MI samples **(B)**, in cluster 1 MI compared with cluster 3 MI samples **(C)**, and in cluster 2 MI compared with cluster 3 MI samples **(D)**.

STRING tools were utilized to predict the PPI of the DEGs. Then, we used Cytoscape MCODE to further screen the core PPI network, and the results revealed four hub networks related to different clusters of MI. In total, 37 nodes and 259 edges were included in the PPI network consisting of DEGs in MI compared with normal samples (Figure 8A), 16 nodes and 120 edges were included in the PPI network consisting with DEGs in cluster 1 MI compared with cluster 2 MI samples (Figure 8B), 55 nodes and 739 edges were included in the PPI network consisting with DEGs in cluster 1 MI compared with cluster 3 MI samples (Figure 8C), and 63 nodes and 982 edges were included in the PPI network consisting with DEGs in cluster 2 MI compared with cluster 3 MI samples (Figure 8D). The module analysis filtered out several genes, including BAG2, BAG3, MMP2, FGF1, HSPB1, CXL12, PJA2, UBE2M, RNF14, RNF16, KLHL2, LTN1, RBM17, LSM2, TRIM37, and UBA3.

### Functional Annotation of Hub Networks Between Different Clusters of Myocardial Infarction

**FIGURE 9:**
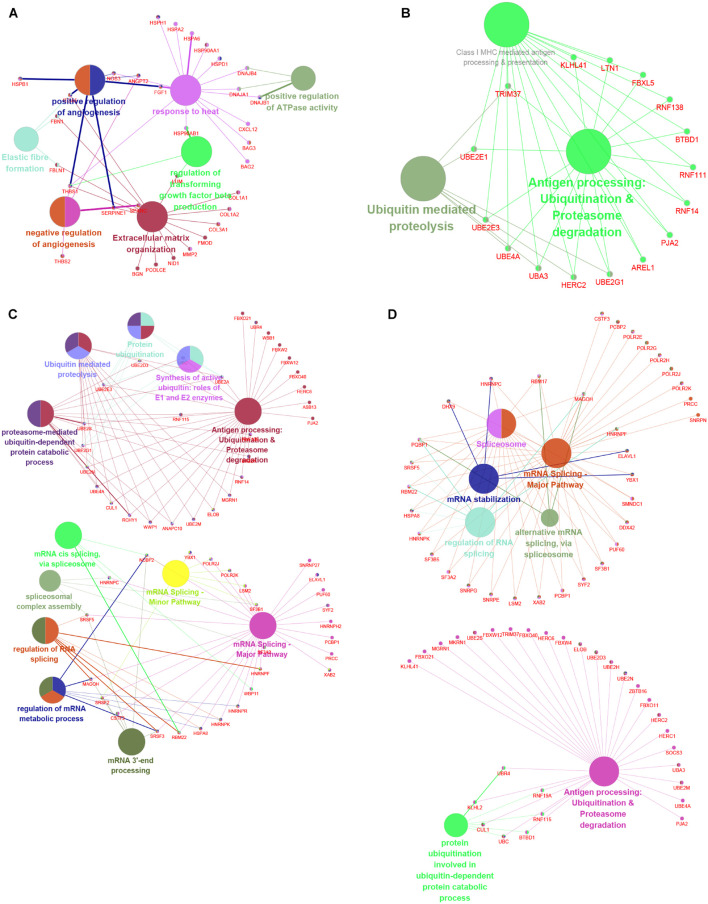
Functional annotation of hub networks in different clusters in MI. **(A–D)** We predicted potential functions of hub networks based on DEGs in MI compared with non-failing controls **(A)**, in cluster 1 MI compared with cluster 2 MI samples **(B)**, in cluster 1 MI compared with cluster 3 MI samples **(C)**, and in cluster 2 MI compared with cluster 3 MI samples **(D)**.

To understand the potential functions involved in the hub networks and the connection between hub genes and biological functions, we performed a ClueGO analysis of these hub networks in MI. As present in Figure 8, the hub PPI network consisting of DEGs in MI compared with normal samples was significantly related to positive and negative modulation of angiogenesis and positive modulation of ATPase activity, response to heat, and extracellular matrix organization (Figure 9A). Of note, we observed multiple HSP family genes, such as HSPH1, HSPA2, HSPA6, HSP90AA1, and HSPD1, were related to MI progression by modulating response to heat. The hub PPI network consisting of DEGs in cluster 1 MI compared with cluster 2 MI samples was significantly related to antigen processing and ubiquitin-mediated proteolysis (Figure 9B). Also, the hub network consisting of DEGs in cluster 1 MI compared with cluster 3 MI samples was involved in regulating multiple biological processes related to protein ubiquitination and RNA splicing (Figure 9C). Finally, the hub network consisting of DEGs in cluster 2 MI compared with cluster 3 MI samples was involved in regulating antigen processing and RNA splicing and stabilization (Figure 9D). Interestingly, we found that C3 genes were significantly related to RNA splicing and stability, consistent with the crucial roles of m6A in RNA splicing and stability. These results indicated that the abnormal regulation of RNAs splicing and stability might contribute to the development of MI.

## Discussion

MI is still the leading threat to human health. m6A is considered one of the most common and abundant RNA methylation modifications in eukaryotes (Wang et al., 2014; Mathiyalagan et al., 2019; Wen et al., 2019). m6A is regulated by m6A methyltransferases and demethylases and controls the fate of target mRNA by affecting splicing, translation, and decay. Recently, some researches suggested that m6A modification exhibits importantly in the development of cardiac remodeling and cardiomyocyte contractile function (Dorn et al., 2019; Huang et al., 2019; Han et al., 2021; Wang et al., 2021). For example, ablation of METTL3 weakened MI-caused myocardial fibrosis by impeding the activation of cardiac fibroblasts (Dorn et al., 2019). WTAP promotes MI *via* modulating ATF4 (Wang et al., 2021). ALKBH5 mediated the modulation of heart regeneration *via* demethylating YTHDF1 mRNA (Han et al., 2021). However, there was lacking the comprehensive analysis of the roles of m6A in MI. In this study, we observed that the levels of FTO, YTHDF3, ZC3H13, and WTAP were dramatically differently expressed in MI tissues compared with non-failing controls, further demonstrating the crucial roles of m6A regulators in MI, which were consistent with previous reports. For example, FTO gene polymorphisms were related to HDL cholesterol concentration and high risk of CVD (Franczak et al., 2018) and could predict the incidence of CVD (Aijala et al., 2015). We also predicted the potential signaling related to m6A regulators and observed that FTO and IGFBP2 are mainly related to glucose measurement, elevated blood glucose level, and chronic schizophrenia. Of note, multiple previous reports had indicated the regulation among FTO and glucose metabolism (Khan et al., 2018; Mizuno, 2018). For example, GWASs showed that FTO mutation is related to impaired fasting glucose (Khan et al., 2018). In the liver, FTO regulated glucose and lipid metabolism, which was modulated by metabolic signals (Mizuno, 2018). HNRNPA2B1 and HNRNPC are mainly related to Astler–Coller B1 rectal carcinoma. IGF2BP3 was related to soft tissue neoplasms and peritoneal neoplasms. Next, we analyzed GWAS data and single-cell sequencing data to confirm the functional importance of m6A regulators in MI. The SNP annotation of the locus found that the m6A regulatory gene WTAP was located in the causal region on chromosome 6, suggesting it may be an important pathogenic gene of MI. Furthermore, we analyzed the single-cell sequencing of the heart tissues and found that WTAP is widely expressed in the heart tissues, whose expression in endothelial cells is the highest. By combining the analysis mentioned earlier that WTAP was significantly lower in the MI group, this suggests that maintaining the normal expression of WTAP may be a prevention or improvement means for the treatment of MI. Very interestingly, our findings were consistent with a recent report. Wang et al. (2021) reported that WTAP promotes MI *via* modulating ATF4. This study, for the first time, reveals novel clues to understand the mechanism of MI based on m6A expression.

The general definition of MI usually consists of five subtypes. In this study, we conducted consensus clustering for MI in view of the dysregulated m6A RNA methylation regulators expression in MI. According to m6A regulators’ expression levels, we clustered 499 MI patients into three subtypes. Our findings demonstrated that FTO, YTHDF1, IGF2BP3, RBM15B, YTHDF3, IGF2BP2, and RBM15 were mostly upregulated in cluster 3. WTAP, HNRNPC, YTHDF2, YTHDC2, HNRNPA2B1, METTL3, ZC3H13, and YTHDC1 were most significantly upregulated in cluster 1. Also, WTAP and HNRNPC were significantly downregulated in cluster 3. Furthermore, our results showed that the DEGs in MI were significantly enriched in multiple signalings, such as the chemokine signaling pathway, calcium signaling pathway, and chemokine signaling. Interestingly, Gene Set Enrichment Analysis further demonstrated that DEGs in MI were related to transport activity and calcium signaling. Moreover, by analyzing the relationship among the top 5 enriched signaling, calcium signaling and chemokine signaling play a key role in MI. Calcium (Ca^2+^), as a second messenger, played a key role in regulating cell proliferation, apoptosis, and survival (Sukumaran et al., 2021). Previous studies had demonstrated that calcium signaling served as a key regulator of MI (Vassalle and Lin, 2004; Li et al., 2021). Under physiological conditions, calcium signaling can effectively modulate the activity of vasodilation/contraction in vascular smooth muscle cells (Li et al., 2021). However, under pathological conditions, Ca2 + overload induces apoptosis in cardiomyocytes, thus leading to MI (Vassalle and Lin, 2004; Jiao et al., 2019). Over the past decades, multiple chemokines and their receptors were found to regulate MI (Altara et al., 2016; Wang et al., 2019; Liang et al., 2021). For example, CXCR3 and its ligands were reported to be valid biomarkers for HF (Altara et al., 2016). CXCL16 was reported to modulate the inflammatory responses in MI (Liang et al., 2021). CXCR4 blockade was found to induce tissue repair after MI by modulating immune-regulatory function (Wang et al., 2019).

Also, bioinformatics analysis indicated that the DEGs between these clusters in MI were related to modulating multiple signalings, such as PI3K-Akt, MAPK, and cGMP-PKG signalings. It is well-known that the PI3K-Akt signaling pathway is a survival-associated signal transduction pathway, protecting the myocardium from ischemic damage (Okumura et al., 2004; Quan et al., 2014; Cheng et al., 2018). Additionally, previous researches have shown that this signaling pathway is motivated when the estrogen receptor β is chronically upregulated. ERK1/2, JNK, and p38 were three subfamilies of MAPK (Zhang and Liu, 2002). The MAPK signaling pathway was reported to promote NF-κB, triggering additional inflammatory cytokines and causing extra damage in myocardial tissue (Verma et al., 2019). The ERK signaling pathway is currently the widely studied one, and it exhibited an association with a variety of biological processes’ regulation, containing cell survival, growth and death, and inflammation-associated immune responses (Ren et al., 2019). cGMP-PKG signaling was considered to be a therapeutic target for myocardial ischemia–reperfusion injury (Ren et al., 2019). As previously described, some studies have shown that cGMP-PKG signaling exerted an effect on the function of the myocardial endoplasmic reticulum and decreased the level of endoplasmic reticulum stress under stress (Schlossmann and Desch, 2011). Moreover, the downregulated genes in cluster 1 compared with cluster 3 and cluster 2 compared with cluster 3 exhibited a significant relation to multiple neurodegenerative diseases. Of note, previous studies revealed abnormal regulation of m6A is related to neurodegenerative disease. For example, Han et al. (2020) reported that the m6A methylation enhanced the development of Alzheimer’s disease. In addition, Du et al. (2021) revealed that the expressions of m6A regulators correlate with neurodegenerative pathways. These reports, together with our findings, revealed the potential crucial roles of m6A in MI.

In our study, we also investigated changes in gene expression among different clusters in MI based on the expression change of the GSE5406 dataset. As present in Figure 7, we observed that approximately 45.7% of DEGs in MI compared with non-failing samples were differently expressed in more than two different clusters. Venn diagram showed four common DEGs among all clusters in MI, including BAG2, CD74, GOLGA8N, and PDK4. Also, those genes were probably regarded as potential indicators for the prediction and diagnosis of MI. For example, BAG3 belongs to the BAG protein family acting as a chaperone molecular *via* physical interaction with Hsp70, HSPBs. It was shown that mutated BAG3 caused DCM, leading to systolic dysfunction, HF, and myofibrillar myopathy (Sturner and Behl, 2017; Diofano et al., 2020). BAG2 was also a member of the human BAG protein family and is expressed in brown adipose, lung, heart, and other tissues. It is shown that BAG2 presented a highly similar sequence and domain to BAG3, indicating BAG2 could compensate for the loss function upon the absence of BAG3 (Sturner and Behl, 2017). PDH kinases, including PDK1–4 mediated the phosphorylation and inactivation of PDH (Vary and Randle, 1985). It proves that the selectively overexpressed PDK4 in the heart gave rise to an obvious alteration in energy metabolism, including the increase in the utilization of fatty acids and the decrease in carbohydrate consumption with the increased pyruvate concentration. These results suggested that overexpressed PDK4 is sufficient to dramatically bring about the change of substrate utilization in the heart (Zhao et al., 2008). CD74 was reported to mediate the effect of macrophage migration inhibitory factor on inflammation and cell proliferation. Migration inhibitory factor–CD74 agonism supplied a potential treatment for acute myocardial ischemia by enhancing AMPK activation (Miller et al., 2008). These results indicated that targeting these genes may provide novel therapy strategies for MI.

Utilizing the STRING database, we constructed the PPI network. The module analysis filtered out several genes, including BAG2, BAG3, MMP2, FGF1, HSPB1, CXL12, PJA2, UBE2M, RNF14, RNF16, KLHL2, LTN1, RBM17, LSM2, TRIM37, UBA3, etc. Some of these genes in acute MI (AMI) have been reported, indicating that our conclusions were consistent with previous integrated bioinformatics analysis data. For instance, Wei Gong et al. (2018) revealed that trimetazidine could prevent cardiac rupture in AMI mice by inhibiting matrix metalloproteinase-2 (Mmp2) and Mmp9 expression levels, indicating that the MMP family was probably related to cardiac remodeling after AMI. Fibroblast growth factor 1 (FGF1), also named acidic FGF, took part in several physiological processes, e.g., development, morphogenesis, and others (Engel et al., 2006; Beenken and Mohammadi, 2009). Overexpression of FGF1 or intramyocardial infusion of FGF1 mimic could exert cardioprotective effects on MI mice models (Htun et al., 1998). Heat-shock proteins (HSPs) are largely expressed in various cells of the cardiovascular system, including endothelial cells, cardiomyocytes, monocytes, and platelets. Kraemer et al. (2019) for the first time, showed the upregulation and phosphorylation of HSP27 (HSPB1) of human platelets during MI on a cellular level *ex vivo*, showing a typical intracellular translocation pattern. Therefore, HSP27 (HSPB1) phenotype in platelets was a measurable stress response indicator in MI and other acute ischemic events [38,39]. After MI, the increased expression of CXCL12 was observed in the infarct area and served as a homing signal for endothelial progenitor cells (Jiao et al., 2019). To understand the potential functions involved in the hub networks and the connection between hub genes and biological functions, we performed ClueGO analysis of these hub networks in MIs. We found that MI-related network was significantly related to positive and negative regulation of angiogenesis and response to heat. The hub networks in MI clusters were significantly related to antigen processing and ubiquitin-mediated proteolysis, RNA splicing, and stability. These results indicated that the abnormal regulation of RNAs splicing and stability might contribute to the development of MI.

## Conclusion

In summary, we observed levels of FTO, YTHDF3, ZC3H13, and WTAP were dramatically differently expressed in MI tissues compared with non-failing controls, further demonstrating the crucial roles of m6A regulators in MI. Bioinformatics analysis showed that m6A regulators were related to regulating glucose measurement and elevated blood glucose levels. Furthermore, GWAS data analysis showed that WTAP SNP was significantly related to the progression of MI. In addition, the single-cell sequencing of the heart tissues found that WTAP is widely expressed in the heart tissues. By combining the analysis mentioned earlier that WTAP was significantly lower in the MI group, this suggests that maintaining the normal expression of WTAP may be a prevention or improvement means for the treatment of MI. Furthermore, three subtypes with different clinical characteristics were constructed based on m6A regulators’ expression profiles, indicating that they were potential therapeutic strategies for MI patients. This study could provide more information for us to understand the roles of m6A in MI, which may provide novel clues to identify biomarkers for clinical treatment and diagnosis.

## Data Availability Statement

The original contributions presented in the study are included in the article/supplementary material, further inquiries can be directed to the corresponding author/s.

## Author Contributions

BnH and L-HS conceived and designed the project and are responsible for the overall content. YC, XZ, CG, H-WN, FL, JX, and ZW analyzed and interpreted the data. XS, CG, BrH, and BnH prepared the manuscript. XS, YC, XZ, YL, JX, XW and ZC contributed to revising the manuscript. All authors contributed to discussed the results, critically reviewed the manuscript, read and approved the final manuscript.

## Conflict of Interest

The authors declare that the research was conducted in the absence of any commercial or financial relationships that could be construed as a potential conflict of interest.

## Publisher’s Note

All claims expressed in this article are solely those of the authors and do not necessarily represent those of their affiliated organizations, or those of the publisher, the editors and the reviewers. Any product that may be evaluated in this article, or claim that may be made by its manufacturer, is not guaranteed or endorsed by the publisher.

## Footnotes

1
http://geneatlas.roslin.ed.ac.uk/

2
https://david.ncifcrf.gov/

3
www.cytoscape.org/
